# Targeted Fcγ Receptor (FcγR)-mediated Clearance by a Biparatopic Bispecific Antibody*

**DOI:** 10.1074/jbc.M116.770628

**Published:** 2017-01-18

**Authors:** Srinath Kasturirangan, G. Jonah Rainey, Linda Xu, Xinwei Wang, Alyse Portnoff, Tracy Chen, Christine Fazenbaker, Helen Zhong, Jared Bee, Zhutian Zeng, Craig Jenne, Herren Wu, Changshou Gao

**Affiliations:** From the Departments of ‡ Antibody Discovery and Protein Engineering,; § Oncology, and; ¶ Analytical Biotechnology, Medimmune LLC, Gaithersburg, Maryland 20878 and; the ‖ Department of Microbiology, Immunology and Infectious Diseases, University of Calgary, Calgary, Alberta T2N 4N1, Canada

**Keywords:** antibody, antibody engineering, electron microscopy (EM), Fc receptor, IL-6, macrophage, microscopic imaging, pharmacokinetics, protein complex

## Abstract

Soluble ligands have commonly been targeted by antibody therapeutics for cancers and other diseases. Although monoclonal antibodies targeting such ligands can block their interactions with their cognate receptors, they can also significantly increase the half-life of their ligands by FcRn-mediated antibody recycling, thereby evading ligand renal clearance and requiring increasingly high antibody doses to neutralize the increasing pool of target. To overcome this issue, we generated a bispecific/biparatopic antibody (BiSAb) that targets two different epitopes on IL-6 to block IL-6-mediated signaling. The BiSAb formed large immune complexes with IL-6 that can bind Fcγ receptors on phagocytic cells and are rapidly internalized. In addition, rapid clearance of the BiSAb·IL-6 complex was observed in mice while the parental antibodies prolonged the serum half-life of IL-6. Intravital imaging of the liver in mice confirmed that the rapid clearance of these large immune complexes was associated with Fcγ receptor-dependent binding to Kupffer cells in the liver. The approach described here provides a general strategy for therapeutic antibodies with the ability to not only neutralize but also actively drive clearance of their soluble antigens.

## Introduction

mAbs are increasingly used as therapeutics for treatment of diverse diseases (1). However, because of their long half-life, mAbs targeting soluble antigens can act as buffering agents, enabling their target antigens to evade normal clearance mechanisms, thereby necessitating an increased dose to achieve therapeutic efficacy (2). A bispecific antibody (BiSAb)^2^ that targets two unique epitopes on a soluble antigen can alleviate this problem by binding the antigen and generating large immune complexes that can exploit clearance mechanisms such as complement activation and Fcγ receptor (FcγR)-mediated internalization and phagocytosis (3).

IL-6 is a multifunctional cytokine that regulates immune response, hematopoiesis, and inflammation (4). Elevated levels of IL-6 contribute to the pathogenesis of various autoimmune and inflammatory diseases (5). IL-6 is also a growth factor that drives proliferation in several tumor types (5), and high serum IL-6 levels correlate with a negative prognosis (6). Therefore, targeting IL-6 and IL-6R by monoclonal antibodies is a promising therapeutic approach for cancer and autoimmune diseases such as rheumatoid arthritis (7). Although anti-IL-6 antibodies show promise in neutralizing IL-6 activity *in vitro*, the serum levels of injected human IL-6 in mice treated with anti-IL-6 antibody are much higher than in control mice injected with human IL-6 alone because of the anti-IL-6 antibody-mediated buffering effect (8). An ideal therapeutic would overcome this limitation by both neutralizing and clearing its target antigen.

The Fc region of monoclonal antibodies plays a critical role in the clearance of immune complexes by interacting with FcγRs on phagocytic cells or by activating complement by binding C1q (9). Immune complexes bind different subsets of FcγRs on “professional” phagocytes such as neutrophils, monocytes, and macrophages, including Kupffer cells (KCs) (10), and liver sinusoidal endothelial cells (LSECs), which are responsible for the clearance of small immune complexes (SICs) in the liver (11, 12). The fate of antibody-antigen complexes is governed in part by size (13–16). A single Ab bound to Ag results in a monomeric immune complex containing a single Fc. Monomeric immune complexes have weak FcγR interactions and do not significantly bind cells in the reticuloendothelial system, and those that bind are generally recycled and are not efficiently cleared (17–19). Higher-order immune complexes form when there is a polyclonal response to antigen, and each antigen can bind more than one antibody simultaneously (20, 21). In this way, both the antibody and antigen can form branch points in complex formation, and immune complex size is dependent on the concentration of antibody, antigen, and the degree of polyclonality (20–23). Depending on their size, these complexes can be soluble or insoluble (20, 23, 24). SICs bind avidly to FcγRs, triggering intracellular trafficking that leads SICs down a degradative endocytic pathway in KCs or LSECs (12, 14, 17, 25–31). Larger insoluble immune complexes can be deposited in circulatory tissues, leading to a variety of diseases (32, 33), or can be bound by complement and solubilized, ultimately leading to clearance similar to SICs (34, 35). Further, large immune complexes can be particles or bacteria that are coated with antibodies, and these are cleared by phagocytosis in a process also involving FcγR interaction (36, 37).

Approaches to overcome buffering of antibodies to enhance clearance of target antigens have been described (29, 38, 39). Enhanced binding to FcRn (40) or lowering the isoelectric point of an antibody (41) have been shown to improve pharmacokinetic properties, which can result in a reduced dose and improved efficacy. Antibodies can also be engineered to bind to target antigens in a pH-dependent manner (42), whereby binding occurs at neutral pH outside of the cell but antigen is released at the mildly acidic pH of the early endosome. Upon release, the antigen follows the default fluid-phase trafficking pathway to lysosomal degradation, whereas the antibody recycles in an FcRn-dependent manner (42, 43).

Here we report a bispecific antibody that targets two different epitopes on IL-6, forms large BiSAb-Ag complexes, and are rapidly cleared by Kupffer cells upon FcγR-mediated internalization *in vivo*. This strategy is broadly applicable and provides the dual benefits of achieving ligand neutralization and fast clearance *in vivo*, thus potentially improving the therapeutic efficacy of the biparatopic antibody.

## Results

### 

#### 

##### Generation and Characterization of Anti-IL-6 Antibodies

**FIGURE 1.**
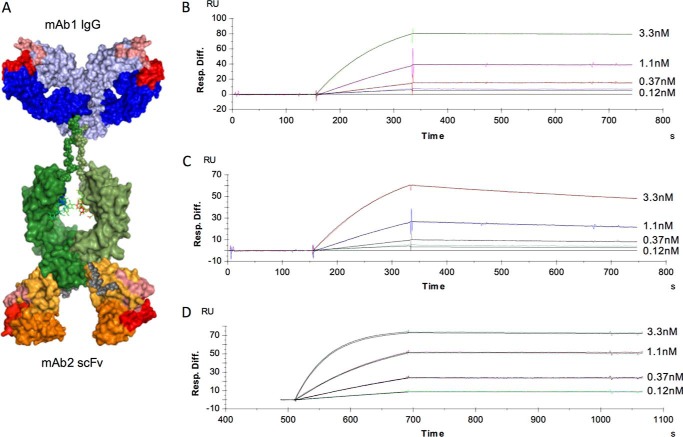
**mAb1, mAb2, and BiS3Ab exhibit high-affinity binding for their antigen, hIL-6.** *A–D*, a bispecific antibody was generated in the BiS3Ab format, with mAb1 as IgG and mAb2 as scFv, where the scFv is attached to the carboxyl terminus of the IgG via a 10-amino acid flexible linker. Fab arms are shown in *blue* (*light blue*, Hc; *dark blue*, Lc), with complementarity-determining regions highlighted in *salmon* (Hc) and *red* (Lc). The hinge and Fc region of the antibody appear in *green*. The scFv is represented in *orange*, with *light orange* representing Hc and *dark orange* representing Lc (*A*). Biacore analysis of mAb1 (*B*), mAb2 (*C*), or BiS3Ab (*D*) was performed with the antibodies immobilized and soluble hIL-6 used as analyte.

Two anti-IL-6 antibodies, mAb1 and mAb2, target two different epitopes on IL-6. BiS3Ab was generated (Fig. 1*A*), where the scFv of mAb2 was appended to the C terminus of the mAb1 Fc joined by a short synthetic linker (44, 45). The antibodies and recombinant human (rh) IL-6 were expressed in HEK293F cells, purified by protein A or nickel-nitrilotriacetic acid, respectively, and polished to >97% monomer by SEC. mAb1 and mAb2 exhibit high binding affinities to rhIL-6 of 297 pm and 12.9 pm, respectively (Fig. 1, *B* and *C*). BiS3Ab also demonstrated a high apparent affinity for rhIL-6 (5.15 pm); however, this is not a true affinity measurement because of the presence of anti-IL-6 variable domains with two different affinities on the same molecule (Fig. 1*D*).

**FIGURE 2.**
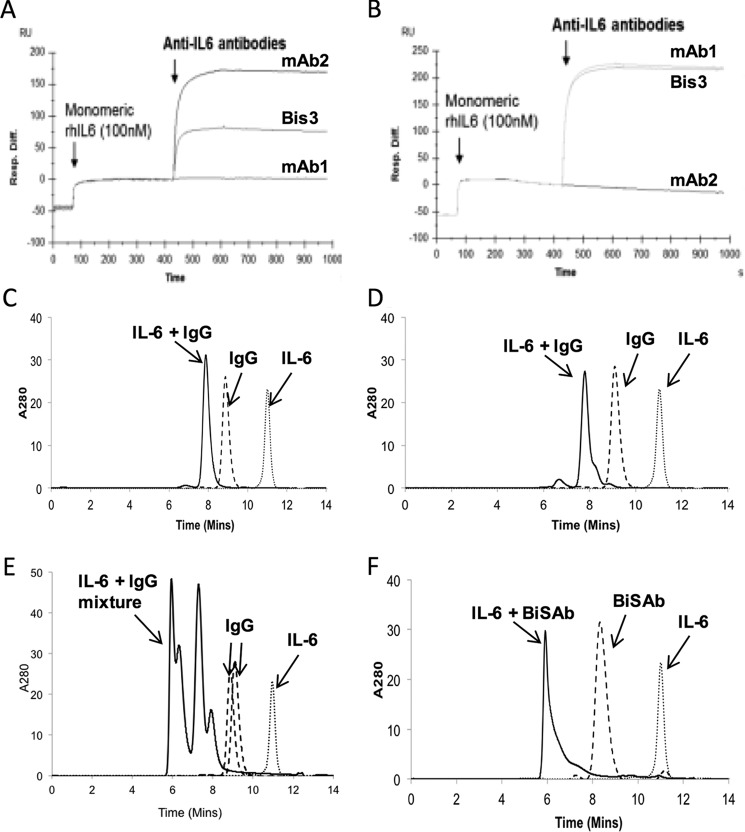
**BiS3Ab can bind both epitopes of IL-6 simultaneously, and the mAb/BiSAb format determines the order of complex formation.** *A* and *B*, mAb1 (*A*) or mAb2 (*B*) was immobilized on a CM5 BIAcore chip. rhIL-6 was captured by the immobilized mAbs, followed by mAb1, mAb2, or BiSAb3. *C–F*, mAb2 (*C*), mAb1 (*D*), a mixture of the two mAbs (*E*), or BiS3Ab (*F*) was mixed with rhIL-6 in a molar ratio of 1:1 IL-6:antibody variable domain to generate immune complexes. The complex generated upon incubation was analyzed by HPLC-SEC.

Concurrent binding was established using Biacore. Anti-IL-6 mAb1 and mAb2 were immobilized; when rhIL-6 antigen was applied to the chip, binding was observed regardless of which parental mAb was bound. Application of the second antibody or BiS3Ab increased the signal, indicating concurrent binding. As expected, subsequent application of the same antibody to the chip did not show any additional binding to the rhIL-6 (Fig. 2, *A* and *B*).

##### Oligomeric Complex Generation Was Observed with BiS3Ab

Next, we created a series of immune complexes with rhIL-6 (mAb1 + IL-6, mAb2 + IL-6, mAb1 + mAb2 + IL-6, or BiS3Ab + IL-6) to generate complexes in a molar ratio of 1:1 of paratope: epitope and analyzed them by HPLC-SEC (Fig. 2, *C–F*).

As expected, given their predicted stoichiometric association, mAb1 and mAb2 showed a shift in the peak corresponding to binding of the antibody to one or two IL-6 molecules. The mixture of two mAbs complexed with rhIL-6 resulted in the generation of complexes corresponding to a range of sizes, including higher-order complexes. This is likely due to some stoichiometric complexation as well as some cross-linking of Ab/Ag interaction resulting in immune complex formation. Strikingly, the complexes generated with BiS3Ab eluted in the void volume, indicating the formation of large oligomeric complexes with a size of >500 kDa. Given the ability of the BiS3Ab to drive formation of larger immune complex and the persistence of large amounts of stoichiometric complex in the mAb1 + mAb2 mixture sample, BiS3Ab was prioritized for further study, and the mixture was not investigated further.

##### Transmission Electron Microscopy Confirms Large Complex Formation

To further characterize the size of the complexes, we performed negative stain EM. A particle size distribution analysis was performed, and the area equivalent diameter (AED) was calculated.

**FIGURE 3.**
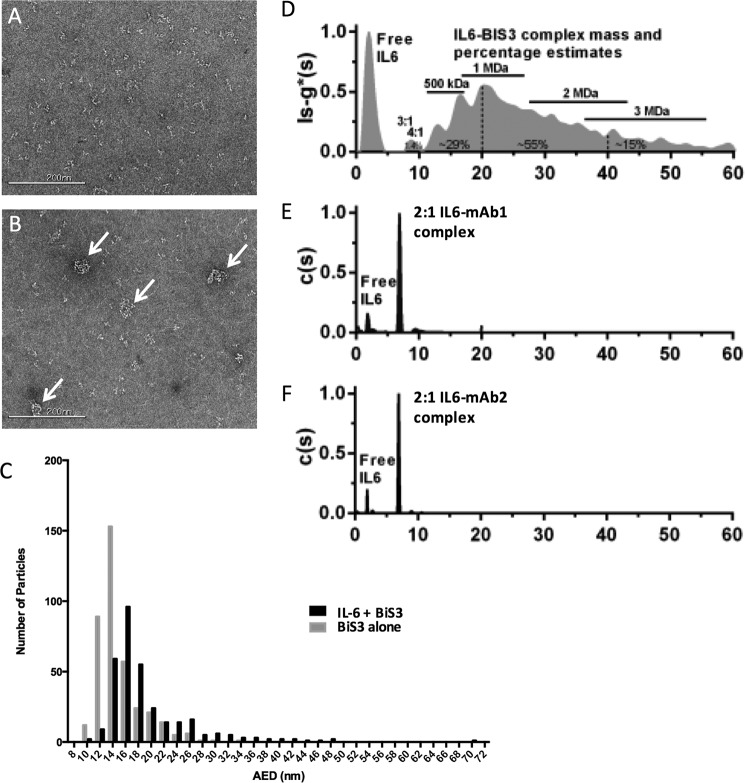
**Negative stain electron microscopy and analytical ultracentrifugation demonstrate the formation of large complexes by BiSAb3 in the presence of IL-6.** *A* and *B*, negative stain EM analysis was performed on the BiS3Ab alone (*A*) or Bis3Ab mixed with IL-6 (*B*). *Scale bar* = 200 nm. *C*, mean AED frequency distribution histogram for the samples. More accurate sizing of complexes by analytical ultracentrifugation defines complex size and predicts stoichiometry. *D*, a broad range of large aggregates formed between rhIL-6 and BISAb3. The approximate mass ranges and percentages of the complexes described in the text are noted as annotations. *E* and *F*, rhIL-6 + mAb1 (*E*) and rhIL-6 + mAb2 (*F*) exhibit only stoichiometric complexes and no large aggregated species. Excess free rhIL-6 was seen at about 2S in all samples.

BiS3Ab alone (Fig. 3*A*) had an AED between 12–16 nm, consistent with a monomer. By contrast, when the BiS3Ab was mixed with IL-6 in a 1:1 epitope:paratope ratio, larger complexes that sequestered most of the antibody and the IL-6 could be observed by TEM (Fig. 3*B*). Complex particle sizes ranged from 14–48 nm AED (Fig. 3*C*), consistent with formation of a majority of complex containing at least three BiSAb molecules. Although this result clearly demonstrates that a large amount of IL-6 is sequestered in larger complexes, it is difficult to interpret what proportion of IL-6 is sequestered, and the stoichiometry of the complexes present.

##### Analytical Ultracentrifugation (AUC) Provides Greater Resolution on Complex Distribution

AUC was used to more precisely characterize complex size. The Svedberg equation can be used to estimate the sedimentation coefficient based on the mass and shape of a complex and determine complex size stoichiometry (46). Fig. 3*D* shows the sedimentation coefficient distribution of the complexes formed between IL-6 and BiS3Ab compared with the corresponding distributions for complexes with mAb1 (Fig. 3*E*) and mAb2 (Fig. 3*F*). The two parental antibodies formed uniform complexes with IL-6, with excess IL-6 remaining in solution. The IL-6-mAb1 and IL-6-mAb2 complexes had estimated masses of 200 kDa and 194 kDa, respectively, as expected for a 2:1 IL-6 to mAb complex. In sharp contrast, the distribution profile for the complexes between IL-6 and the BiS3Ab clearly showed the formation of a broad range of large complexes. Although only a small amount of complex comprised of a single BiS3Ab bound to three or four IL-6 molecules could be detected, accounting for only 1.4% of the total complexes, complexes with two BiS3Ab molecules covering a mass range of about 420–560 kDa represented about 29% of the total complexes in solution. Larger oligomeric complexes that contained at least three BiS3Ab molecules with a size range between 0.5 to 3 MDa represented about 70% of the complexes and were the predominant species in the mixture.

##### Complex Generated with BiS3Ab Showed Enhanced Binding to Human FcγRs

**FIGURE 4.**
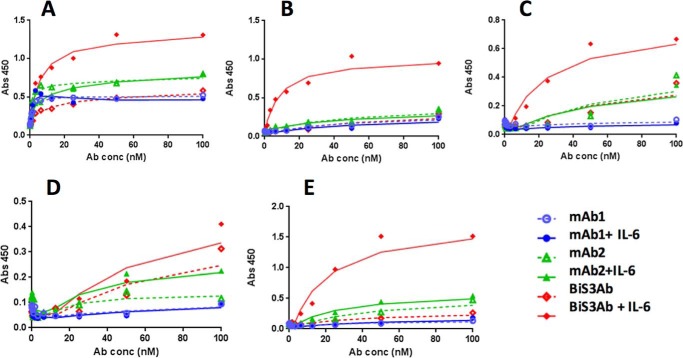
**Complex formation enhances binding to FcγRs.** *A–E*, binding of Ab-Ag complex generated by the parental mAbs or BiS3Ab with rhIL-6 to immobilized hFcγRs: hFcγRI (*A*), hFcγRIIA (*B*), hFcγRIIb (*C*), hFcγRIIIA-158F (*D*), and hFcγRIIIA-158V (*E*) was determined by ELISA.

Binding of Ab-Ag complex to a panel of human FcγRs consisting of FcγRI, FcγRIIa, FcγRIIb, and both the lower-affinity 158F and higher-affinity 158V alleles of FcγRIIIa were determined by ELISA (Fig. 4). The individual mAbs or mAbs incubated with rhIL-6 showed weak binding to all FcγRs tested. The higher-order oligomeric species generated by incubating rhIL-6 with BiS3Ab bound strongly to FcγRs I, IIA, IIB, and IIIA. Binding of Ab-Ag complex to mouse FcγRs was confirmed by Octet analysis (data not shown).

##### Complexes Generated by BiS3Ab Were Phagocytosed Rapidly in Vitro

**FIGURE 5.**
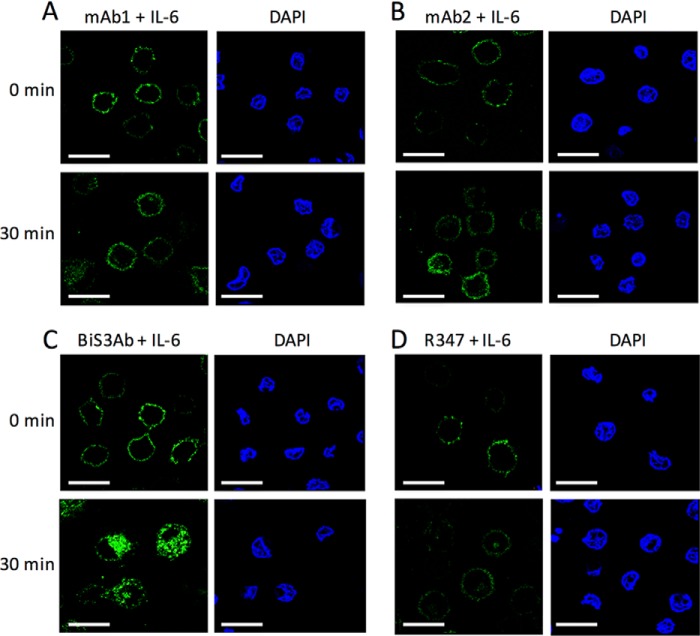
**Induced macrophage-like cells phagocytose BiSAb·Ag complexes.** *A–D*, the Ab-Ag complexes generated with AF488-labeled (*green*) parental antibody alone (*A* and *B*), BiS3Ab (*C*), or an isotype control R347 (*D*) were added to PMA-stimulated U937 cells. After 30-min incubation at 37 °C, most of the signal for the parental mAbs incubated with rhIL6 remained on the surface (*A* and *B*), whereas the complex generated by incubating rhIL6 with BiS3Ab was shown to be rapidly internalized into the cells (*C*), confirming phagocytosis of the larger complexes. DAPI staining (*blue*) shows the nuclei of the cells in each field. *Scale bars* = 50 μm.

Having established complex-dependent association of BiS3Ab with purified FcγRs, we wanted to demonstrate that complexes could bind and internalize into cells expressing these receptors. Ab-Ag complexes generated by incubating AF488-labeled antibodies with rhIL-6 were added to PMA-stimulated U937-induced macrophage-like cells and incubated on ice to allow binding while preventing internalization. When cells were subsequently shifted to 37 °C for 30 min, most of the signal for the mAbs alone or bound to rhIL-6 remained on the cell surface, similar to the background staining observed with a nonspecific antibody, R347, indicating that there was no internalization. In contrast, the larger oligomeric rhIL-6 + BiS3Ab complexes were internalized rapidly, with ∼50% internalized within 30 min, consistent with active FcγR-mediated phagocytosis (Fig. 5).

##### Complexes Generated by BiS3Ab Were Cleared Rapidly in Vivo

**FIGURE 6.**
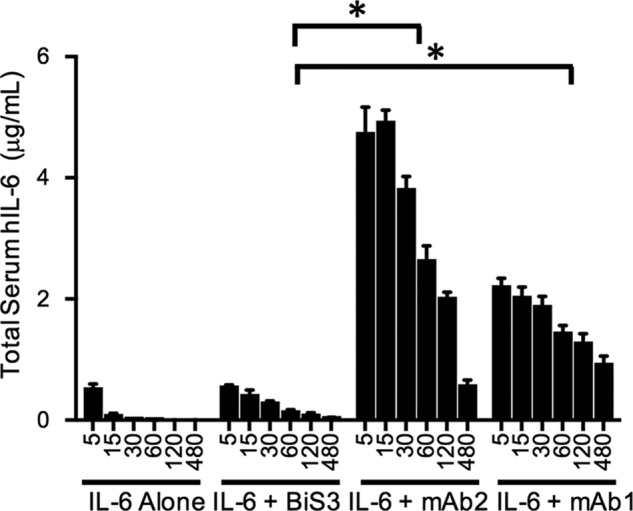
**BiS3Ab/IL6 complex is efficiently cleared *in vivo*.** Mice were injected with rhIL-6 alone or rhIL-6 incubated with the individual mAbs or BiS3Ab. The rhIL-6 antigen alone has a short half-life and is cleared rapidly. However, when rhIL-6 was incubated with the parental mAbs, it persisted in the circulation because of the buffering effect of the mAbs. The oligomeric complex generated by incubating rhIL-6 with BiS3Ab was cleared rapidly *in vivo*. *, *p* < 0.005 for group-to-group comparison.

To determine whether the *in vitro* binding and phagocytosis of the BiS3Ab-IL-6 complex would translate into fast *in vivo* clearance, mice were injected with rhIL-6 alone or rhIL-6 incubated with the mAbs or BiS3Ab. rhIL-6 was cleared rapidly in mice, with only a small amount detectable 5 min after injection. As predicted for stoichiometric Ab-Ag complexes that bind FcRn, the serum half-life of rhIL-6 bound to parental mAbs was prolonged considerably (Fig. 6). In contrast, rapid clearance comparable with rhIL-6 alone was observed with the oligomeric complexes generated by BiS3Ab. Interestingly, a small amount of rhIL-6 persisted at the 1-h time point, consistent with the stoichiometric complexes detected for BiS3Ab·rhIL-6 as observed by AUC.

##### Large Oligomeric Complexes Formed by BiS3Ab Accumulated in the Liver

**FIGURE 7.**
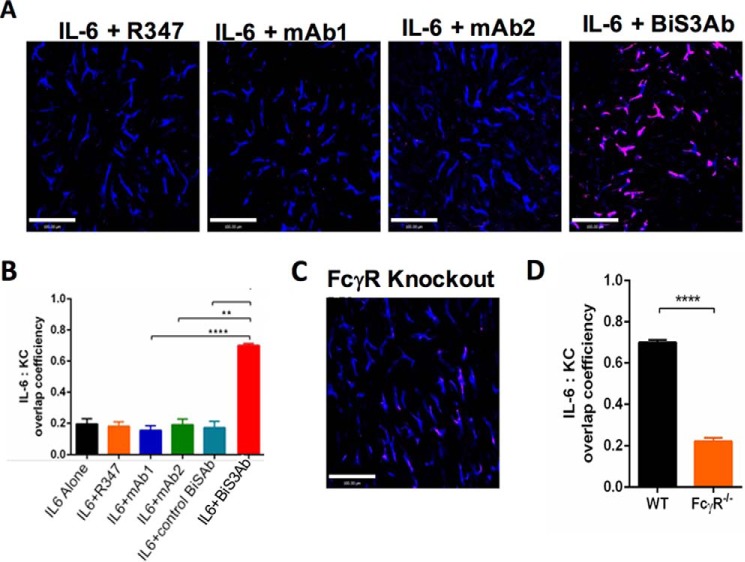
**Clearance of immune complex takes place in the Kupffer cells in the liver.** WT mice were injected intravenously with rhIL-6 + R347 (isotype control), rhIL-6 + mAb2, rhIL-6 + mAb1, or rhIL-6 + BiS3Ab. Intravital imaging of the liver was conducted immediately following injection. *A*, representative images of rhIL-6 capture 20 min after injection for each group. *B*, the co-localization efficiencies per field of view between red and blue signals in each group. *C*, representative image of Fcϵr1^−/−^ mice injected with rhIL-6 + BiS3Ab. *D*, the co-localization efficiencies of rhIL-6 + BiS3Ab complex within Kupffer cells injected in WT and Fcϵr1^−/−^ mice were compared. For *A* and *C*, *red* indicates IL6 and *blue* indicates F4/80. *Scale bars* = 100 μm. Data are expressed as mean ± S.E., *n* = 3–6 mice/group. For each mouse, three to six random fields of view were analyzed. **, *p* < 0.01; ****, *p* < 0.0001.

The liver is the primary site of clearance for a variety of pathogens and antigenic complexes (47–49). To determine whether the complexes generated by BiS3Ab accumulate in the liver of mice, intravital microscopy (IVM) was employed. This technique allows the direct visualization and cellular localization of fluorescently labeled immune complexes in living mice. Monomeric rhIL-6 or rhIL-6 mixed with the parental antibodies showed no liver localization, with the signal intensity similar to that of the isotype control. A strikingly more intense signal was observed when immune complexes generated by BiS3Ab were injected, with about 70% of the signal co-localized with KCs, indicating that these large oligomeric complexes associate with these cells (Fig. 7, *A* and *B*). FcϵR1γ ^−/−^ mice were used to explore the role of FcγRs in liver accumulation of IL-6-containing complexes *in vivo* (50). This common γ chain is a signaling component for FcγR1, 3, and 4 in mice, with FcγRI expression reduced by 80% in knockout mice (51). Interestingly, in these mice, there was no accumulation of the BiS3Ab·IL-6 complex within the liver (Fig. 7, *C* and *D*), demonstrating that the liver accumulation and clearance of the large immune complexes in mouse liver KCs is FcγR-mediated.

## Discussion

Targeting soluble ligands for therapy has led to many transformative medicines, but the buffering effects of an antibody prolonging the half-life of bound antigens complicates inhibitory mechanisms (8). BiSAbs can be used to target two different antigens or two different epitopes on the same antigen (52). Previously, we and others have reported strategies to generate bispecific antibodies by appending single-chain Fv (scFv) fragments at various locations on an IgG (44, 45).

Biparatopic bispecific antibodies that bind two epitopes on the same soluble antigen and form immune complexes have been reported previously (53, 54). These complexes bound FcγRs avidly and induced phagocytosis and degradation *in vitro* (53). Further, when a BiSAb was administered to cynomolgus monkeys, although complex formation was observed *in vitro*, there was not any measurable decrease in target antigen concentration, most likely because of the limited complex sizes of two to three BiSAbs observed (54). However, the size of complexes generated in this previous study was limited to dimers and trimers and not the larger-sized complexes reported here.

Here we have demonstrated that BiSAbs that target two distinct epitopes on IL-6 drive the formation of large complexes. The majority of these complexes contain three or more BiSAb molecules and, therefore, would be expected to drive avid Fc-FcγR interaction with phagocytic cells. Consistent with this expectation, these complexes bind FcγRs *in vitro* and also bind and are internalized into induced macrophages. These data support a model in which complex formation drives FcγR binding and internalization *in vivo*, leading to rapid clearance resulting from uptake by phagocytic cells in the liver. Consistent with this model, BiSAb/IL-6 complexes are indeed cleared rapidly in mice and accumulate in the liver, co-localizing with resident macrophages, Kupffer cells.

Datta-Mannan *et al.* (11) have reported that bispecific antibodies inherently exhibit rapid clearance in monkeys. Whether this is a general principle remains to be determined. They reported that BiSAbs are not associated with Kupffer cells but, instead, associated with LSECs. Here we report that BiS3Ab·IL-6 complexes localize to Kupffer cells, clearly differentiating the BiS3Ab·IL-6 complex clearance mechanism from that reported by Datta-Mannan *et al.* (11). Ongoing and future work will elucidate the role of the BiSAb format in pharmacokinetic behavior and will be the subject of a separate report. However, the sum of the data presented here suggests that clearance of BiS3Ab·IL-6 complexes is driven by complex formation itself and not the molecular format of the BiSAb.

Here we have presented a proof-of-concept study demonstrating that rapid clearance of soluble antigens can be driven by forming immune complexes that contain three or more BiSAb molecules. For the proof of concept, we maintained a 1:1 ratio; however, a therapeutic BiSAb designed to drive clearance would present a more complicated dynamic. To conceptualize complex sizes and clearance behaviors, it is useful to examine the limited cases of excess antigen on one hand and excess BiSAb on the other. With excess antigen, all binding sites on each BiSAb molecule would be occupied by single antigens. In this case, a stoichiometry of 1:4 BiSAb:Ag would be achieved, and negligible amounts of larger immune complex would form. The clearance of antigen bound to BiSAbs would be attenuated, but the majority of antigen would follow its normal metabolic path because it would not be bound to BiSAbs. As BiSAb concentration increased, larger complexes would form. Although it is not clear at what ratio and concentration a maximum would occur, we have experimentally demonstrated that a 1:1 ratio drives very efficient complex formation under the conditions we tested. At excess BiSAb, antigen would become limiting, each antigen molecule would interact with a single BiSAb, and a stoichiometry of 1:1 BiSAb:Ag would be achieved. Under these circumstances, the BiSAb would efficiently prolong the half-life of the entire accessible pool of antigen. This thought experiment demonstrates the complexity of implementing the strategy proposed here *in vivo*. Further studies are needed to elucidate parameters governing the “clearance equivalence zone” in terms of BiSAb:Ag ratio, concentration, and affinity. Additionally, it is possible that, in local environments such as a tumor or inflamed tissue, the antigen concentration may be higher, and BiSAbs may form complexes under these conditions, selectively driving clearance from diseased areas in the body.

We describe a biparatopic bispecific antibody that can provide the dual benefits of ligand neutralization and generation of large oligomeric complexes that rapidly accumulate in the liver and are cleared by an FcγR-dependent mechanism. This dual strategy of neutralization and rapid clearance of target antigen overcomes issues of antibody-mediated antigen buffering and can lead to the development of superior antibody-based therapeutics against soluble targets.

## Experimental Procedures

### 

#### 

##### Generation of BiSAbs

mAb1 and mAb2 were derived from phage libraries and bound different epitopes of IL-6. MAb2 scFv was synthesized in the variable heavy-variable light (VH-VL) orientation with a (G4S)_4_ linker (GeneArt, Life Technologies). The bispecific antibody was generated in the BiS3Ab format (Fig. 1) with the mAb2 scFv appended to the C terminus of the mAb1 IgG using standard molecular biology protocols as described previously (44, 45). The bispecific antibody gene was cloned into a CMV promoter-driven expression vector bearing EBNA-1 and oriP. For antibody expression, 200 μg of DNA was transfected into 300 ml of 1 × 10^6^ cells/ml HEK293F suspension cells with 293Fectin reagent (Invitrogen) according to the protocol of the manufacturer. The cell culture volume was doubled on days 3 and 6, and supernatant containing secreted BiS3Ab was harvested on day 10. Expression titers were determined using analytical scale protein A HPLC (Agilent). BiS3Ab was purified using a HiTrap protein A column (GE Healthcare Life Sciences), eluted using IgG elution buffer (pH 2.8, Pierce), neutralized with Tris-HCl (pH 8), and dialyzed into PBS. The purified protein concentration was determined by Nanodrop Spectrophotometer ND-1000, and the monomer content of BiS3Ab was determined by size exclusion chromatography (SEC) using a TSK-GEL G3000SWXLcolumn (Tosoh Bioscience LLC, Montgomeryville, PA). Aggregates were removed using a Superdex 200 column (GE Healthcare Life Sciences).

##### Generation of rhIL-6

The rhIL-6 gene (Uniprot P05231) was synthesized by GeneArt, Life Technologies and cloned into a CMV promoter-driven expression vector bearing EBNA-1 and oriP with a His_10_ tag. rhIL-6 was expressed in HEK293F cells and purified on a HiTrap nickel column (GE Healthcare Life Sciences). Protein was eluted with 250 mm imidazole and dialyzed into PBS. Aggregates were removed using a Superdex 200 column (GE Healthcare). Carrier-free rhIL-6 for use in *in vivo* experiments was purchased from R&D Systems (Minneapolis, MN).

##### Binding to rhIL-6

Binding affinities of mAbs or BiS3Ab to rhIL-6 were determined with a BIAcore 3000 instrument (GE Healthcare Life Sciences). 100 nm mAb1, mAb2, or BiS3Ab was immobilized on a CM5 chip, and 0.12 nm, 0.37 nm, 1.1 nm, or 3.3 nm rhIL-6 was applied to the chip. Concurrent binding of mAbs and BiSAb to rhIL-6 was demonstrated by BIAcore 3000 instrument (GE Healthcare Life Sciences). 100 nm mAb1 or mAb2 was immobilized on a CM5 chip, and 100 nm rhIL-6 was applied to the chip. Upon stabilization, the same antibody, the second antibody, or BiS3Ab (100 nm) was injected to determine concurrent binding. Experiments were performed at 25 °C in 50 mm sodium phosphate buffer (pH 6), 150 mm NaCl, and 0.05% Tween 20. Data were analyzed using BIAevaluation 4.1 (GE Healthcare Life Sciences), and GraphPad Prism was used to plot the data.

##### Generation and Characterization of Immune Complex

The individual mAbs, a mixture, or BiS3Ab were diluted in PBS (pH 7.4) to a final concentration of 2.5 μm, mixed with monomeric rhIL-6 in a 1:1 molar ratio of paratope:epitope, and incubated for 5 min at room temperature. HPLC-SEC (Agilent 1100 capillary LC system) using a TSK-GEL G3000SWXL column (Tosoh Bioscience LLC) was used to characterize Ab-Ag complexes. The mobile phase was 100 mm sodium phosphate (pH 6.8). For sizing, a low molecular weight gel filtration calibration kit (Bio-Rad) was used.

##### Transmission Electron Microscopy (TEM) and Image Analysis

Negative stain electron microscopy for the examination of Ag-Ab complex was performed at Nanoimaging Services (San Diego, CA). Samples were prepared on continuous carbon films supported on nitrocellulose-coated 400 mesh copper grids (Ted Pella). A 3-μl sample was applied to a freshly plasma-cleaned grid for 1 min and blotted to a thin film and then stained with 2–3% (w/v) uranyl formalyn or uranyl acetate and air-dried. TEM was performed using an FEI Tecnai T12 electron microscope with an FEI Eagle 4k × 4k charge-coupled device (CCD) camera. Images were acquired at nominal magnifications of ×110,000, ×67,000, and ×52,000 using Leginon at a nominal underfocus of −2 μm to −4 μm and electron doses of ∼25–45 electrons/Å^2^.

Images were processed with Appion (55), and contrast transfer functions were corrected using Ace2 (56). Individual particles in the ×67,000 images were selected using automated picking, followed by several rounds of reference-free alignment and classification based on XMIPP (57).

##### Analytical Ultracentrifugation

Complexes of IL-6 and BiS3Ab or control mAbs were prepared at an epitope-to-paratope ratio of 1:1 in PBS (pH 7.4). Samples and buffer were filled into 12-mm double-sector cells with Epon centerpieces and sapphire windows in an An50-Ti rotor in a Beckman ProteomeLab centrifuge at 20 °C, and interference scans were collected at 42,000 rpm at a radial resolution of 0.002 cm over the range 5.9–7.2 cm. Sedfit was used to generate distributions from the raw data as described previously (58). For the complexes between control antibodies and IL-6, the continuous c(s) distribution analysis was used with one discrete component to account for sedimentation of a buffer species at about 0.21S. Data were fit using ls-g*(s) analysis (59). For complexes with control antibodies, a resolution of 0.5–20S at 0.1S was used. For large aggregate complexes, a range of 0–80S was modeled at a resolution of 0.2S.

##### Binding of Immune Complex to FcγRs

The FcγRs RI, RIIA, RIIB, RIIIA (158V), and RIIIA (158F) were produced by MedImmune and coated on high binding Costar^TM^ 96-well plates (Fisher Scientific) at a concentration of 2 μg/ml in PBS (pH 7.4) at 4 °C overnight. Immune complexes were prepared at a final antibody concentration of 15 μg/ml. 3-fold dilutions were made in PBS (pH 7.4), added to wells, and incubated for 2 h at 25 °C. Bound mAb or BiSAb was detected with goat anti-human Fc-HRP (Abcam, San Francisco, CA) using 3,3′,5,5′-tetramethylbenzidine (TMB) reagent (Sigma). Data were analyzed using GraphPad Prism.

##### Phagocytosis Assay

The differentiated human leukemic monocyte lymphoma cell line U937 has been used extensively to measure FcγR-mediated internalization and clearance of immune complexes *in vitro* (60). Upon stimulation with phorbol 12-myristate 13-acetate (PMA, Sigma), U937 cells can be induced to mature and differentiate, adopting the morphology and characteristics of mature macrophages expressing FcγRs (61). U937 cells were seeded in 12-well plates with glass coverslips placed in each well in growth medium containing 10 μg/ml PMA for 48 h. Cells were incubated with AF488-conjugated Ab·Ag complexes on ice in 200 μl of PBS containing 0.05% azide (PBSA) for 1 h and transferred to 37 °C for 30 min. Cells were fixed in formaldehyde and reacted with rhodamine-conjugated anti-human IgG (Thermo Fisher Scientific, Waltham, MA) in PBS (pH 7.4). The nuclei of cells were stained with DAPI. Images were visualized using a Leica confocal microscope at 180× magnification.

##### In Vivo Clearance

6 μg of rhIL-6 alone (R&D Systems) or rhIL-6 incubated with mAbs or BiS3Ab in a 1:1 epitope: paratope ratio in PBS (pH 7.4) was injected i.v. into mice in triplicate. Mouse plasma was collected 5, 15, 30, 60, 120, and 480 min after dosing, and the serum IL-6 levels were calculated using a Quantikine IL-6 assay kit (R&D Systems). Data were analyzed and plotted using GraphPad Prism.

##### IVM

Animal experiments were approved by the University of Calgary Animal Care Committee (protocol no. AC14–0054) and conform to the guidelines established by the Canadian Council for Animal Care. Mice were anesthetized by i.p. injection of 200 mg/kg ketamine (Bayer Inc. Animal Health, Toronto, ON, Canada) and 10 mg/kg xylazine (Bimeda-MTC, Cambridge, ON, Canada).

##### Mice

Wild-type C57BL/6 (The Jackson Laboratory, Bar Harbor, ME) and Fcer1γ-deficient (Taconic Farms, Germantown, NY) colonies were maintained in specific pathogen-free facilities at the University of Calgary. Animals were between 7 and 10 weeks of age and weighed 20–30 g.

##### Treatments

IVM of the mouse liver was performed as previously described (62). Briefly, the tail veins were cannulated to permit delivery of Ab and for maintenance of anesthetic. Body temperature was maintained. The mouse was placed in a right lateral position and the liver was externalized onto a glass coverslip on the inverted microscope stage. Exposed abdominal tissues and liver were covered with saline-soaked absorbent material to prevent dehydration. IV administration of 1 μg of eFluor 660-conjugated rat anti-mouse F4/80 (clone BM8) (eBioscience; San Diego, CA) was used to label KCs. rhIL-6 was labeled with Alexa-555 (Life technologies, Gaithersburg, MD). 3 μg of Alexa-555 conjugated IL-6 mixed with mAbs or BiSAbs at an epitope to paratope ratio of 1:1 was injected intravenously into mice 1 min after the beginning of live imaging.

##### Imaging

Resonance-scanning intravital microscopy was performed using a Leica SP8 inverted microscope (Leica Microsystems, Concord, ON, Canada), equipped with a Piezo focus drive and a motorized stage. This microscope is fitted with a motorized objective turret equipped with HC FLUOTAR L 25×/0.95 W VISIR and is mounted to an optical table (Newport, Irvine, CA) to minimize vibration when imaging. This microscope is equipped with 488-, 552-, and 638-nm excitation lasers that were used together with an 8-kHz tandem scan head for the simultaneous excitation of target fluorophores. Fluorescence was visualized through the filter-free acousto-optical beam splitter coupled to a mix of conventional PMT and hybrid HyD detectors. Leica application suite software (v.4.01, Leica Microsystems, Wetzlar, Germany) was used to drive the confocal microscope and for data acquisition.

##### Image Processing and Analysis

Images or videos were exported, processed, and analyzed with Volocity software (v. 6.3, Improvision). The minimum threshold values were adjusted for each of the fluorescence channels to reduce background. The same threshold values were applied to images/videos from all treatment groups. The co-localization ratio between red (IL-6) and blue (KCs) channels was calculated by using the co-localization function of the Volocity software. The total deposition of red (IL-6) signals per field of view was calculated by using the “Find object” function of the Volocity software.

##### Statistical Analysis

Data were expressed as mean ± S.E. and analyzed by an overall one-way analysis of variance with Tukey's test for multiple groups' comparison (Fig. 7*B*) or by unpaired Student's *t* test for comparison between two groups (Fig. 7*D*) (*, *p* < 0.05; **, *p* < 0.01; ***, *p* < 0.001; ****, *p* < 0.0001).

## Author Contributions

S. K., C. J., G. J. R., and C. G. conceived the hypothesis and designed the research activities. S. K., L. X., X. W., A. P., T. C., C. F., J. B., and Z. Z. performed the experiments and analyzed the data. S. K., G. J. R., H. Z., and C. G. interpreted the results and contributed to the writing of the manuscript. C. G. and H. W. funded the project and contributed to critical review of the manuscript.

## Footnotes

2 The abbreviations used are:

BiSAb: bispecific/biparatopic antibody
FcγR: Fcγ receptor
KC: Kupffer cell
LSEC: liver sinusoidal endothelial cell
SIC: small immune complex
Ab: antibody
Ag: antigen
scFv: single-chain Fv
SEC: size exclusion chromatography
AED: area equivalent diameter
TEM: transmission electron microscopy
AUC: analytical ultracentrifugation
PMA: phorbol 12-myristate 13-acetate
IVM: intravital microscopy
rhIL-6: recombinant human interleukin-6.
